# Thermal and immunological stress modulate the locomotor performance of female *Xenopus laevis* frogs

**DOI:** 10.1093/conphys/coag035

**Published:** 2026-06-18

**Authors:** Thaysa G Oliveira, Laurie Araspin, Laura Camila Cabanzo-Olarte, Carlos A Navas, Anthony Herrel

**Affiliations:** Department of Ecophysiology and Evolutionary Physiology, University of São Paulo USP, Institute of Biosciences IB, Rua do Matão 14, São Paulo, SP 05508-090, Brazil; Département Adaptation du Vivant, UMR 7179 C.N.R.S./M.N.H.N., 55 rue Buffon, Paris 75005, France; Department of Ecophysiology and Evolutionary Physiology, University of São Paulo USP, Institute of Biosciences IB, Rua do Matão 14, São Paulo, SP 05508-090, Brazil; Department of Ecophysiology and Evolutionary Physiology, University of São Paulo USP, Institute of Biosciences IB, Rua do Matão 14, São Paulo, SP 05508-090, Brazil; Yale University, EEB/YIBS, New Haven CT, 06520 United States; Département Adaptation du Vivant, UMR 7179 C.N.R.S./M.N.H.N., 55 rue Buffon, Paris 75005, France; Naturhistorisches Museum, Bernastrasse 15, Bern CH-3005, Switzerland; Department of Biology, Evolutionary Morphology of Vertebrates, Ghent University, Ghent 9000, Belgium; Department of Biology, University of Antwerp, Wilrijk 2610, Belgium

**Keywords:** amphibians, climate change, immune challenge, locomotor performance, thermal physiology

## Abstract

Temperature strongly influences physiological performance and immune function in ectotherms, making ectotherms like amphibians particularly vulnerable to the combined effects of climate change and infectious diseases. Since immune activation is energetically expensive, infected individuals may face dilemmas between maintaining immune defences and sustaining ecologically critical functions such as locomotion. These dilemmas are expected to be especially pronounced in females due to their high reproductive investment. In this study, we investigated how a simulated infection affects locomotor performance in female *Xenopus laevis* frogs under three ecologically relevant thermal conditions: cold (15°C), optimal (22°C) and warm (27°C). Frogs were injected with saline solution (control) or lipopolysaccharide (LPS), and locomotor endurance and jump force were measured. Immune activation reduced locomotor performance at all temperatures. Locomotor endurance was highest near the optimum temperature and decreased at both cold and hot extremes, particularly in frogs treated with LPS, while LPS-induced reductions in jump force were similar across all temperatures. These results indicate that extreme temperatures can intensify the functional costs of immune activation, potentially limiting dispersal, predator escape and other behaviours essential for population persistence. Our findings highlight the importance of integrating thermal stressors and disease when assessing amphibian vulnerability under ongoing climate change.

## Graphical Abstract

**Graphical Abstract:**
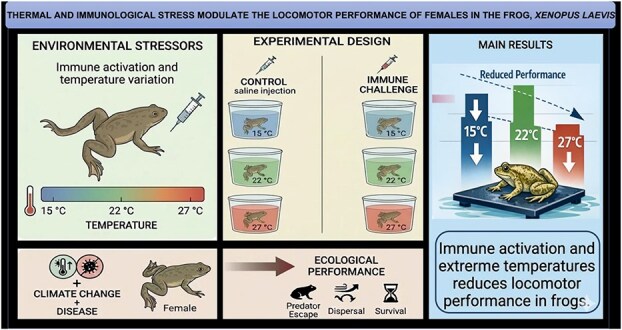

## Introduction

The survival and performance of individuals, as well as the geographic distribution of ectothermic animals, are strongly influenced by abiotic and biotic conditions, which can act as stress factors. Examples include transitions to extreme environmental temperatures (Bartelt *et al.*, 2022) or exposure to pathogens (Hart, 1988; Adelman and Martin, 2009). In amphibians, temperature directly influences the functioning of most physiological systems, impacting metabolic rate and locomotor performance, as well as other ecologically related characteristics, since it determines the ability to forage, escape predators, reproduce and disperse (Huey, 1979; Angilletta *et al.*, 2002; Navas *et al.*, 2008), and respond to infection (Raffel *et al.*, 2006). Therefore, exposure to pathogenic microorganisms, particularly emerging ones, may affect population stability in amphibians, on a global scale, and this impacts relate to climate change scenarios, which may favour both the proliferation of pathogens and exposure to environmental extremes (Pounds *et al.*, 2006; Butler *et al.*, 2013; Seebacher *et al.*, 2014).

In amphibians, environmental temperatures and immune responses are physiologically and ecologically interconnected in several ways. On one hand, the key steps in the immune system are temperature-dependent, including immune cell proliferation, cytokine production and the inflammatory response (Rollins-Smith, 2017; Cohen *et al.*, 2018). On the other hand, behavioural fever, experimentally investigated with lipopolysaccharide (LPS) treatments, leads to the seeking of and/or remaining at elevated temperatures to combat pathogens in some species (Olarte, 2017). LPS is an endotoxin derived from the cell wall of Gram-negative bacteria that induces a systemic inflammatory response, similar to that observed during real bacterial infections, without the need to introduce a viable pathogen (Bicego and Branco, 2002; Adelman and Martin, 2009; Braga, 2013). LPS can also trigger behavioural responses leading to reduced general activity, which has already been observed in some amphibian species (Llewellyn *et al.*, 2011; Oliveira, 2022; Olarte *et al.*, 2024). Therefore, in amphibians LPS injection can trigger behavioural fever, increased inflammatory markers and sickness, a response that generally reduces activity and locomotion (Bicego and Branco, 2002; Braga, 2013; Rollins-Smith, 2017; Oliveira *et al.*, 2024a, 2024b).

Considering that immunological challenges can affect amphibian behaviour and that locomotion is a central component of individual fitness, understanding behavioural responses to infection is relevant. Locomotion determines success in escaping predators, foraging, accessing breeding sites and dispersal—processes critical both for the persistence of natural populations and for the invasive potential of exotic species (Taigen *et al.*, 1982; Pough *et al.*, 1992). Like most ectothermic vertebrates, amphibians exhibit a typical thermal curve for locomotion, with a marked reduction at temperatures below or above the ideal physiological temperature (Huey and Kingsolver, 1989; Navas *et al.*, 2008; Angilletta, 2009; Schulte *et al.*, 2011). Thus, locomotor performance in anuran amphibians varies markedly at low or high temperatures. In cold conditions, limitations associated with the musculature reduce contractile velocity, leading to reduced activity (Huey, 1979; Huey and Kingsolver, 1989; Navas *et al.*, 1999, 2008). In contrast, under elevated temperatures, peak performance can be compromised by systemic constraints, including metabolic limitations and functional integration factors, even when muscle capacity is maintained, leading to rapid declines in locomotor performance (Navas *et al.*, 2008; Tattersall *et al.*, 2012; Seebacher *et al.*, 2014).

These effects become particularly relevant when exposure to extreme temperatures occurs concomitantly with the activation of the immune system. Both low and high temperatures can lead to a decrease or suppression of the immune response, but through different mechanisms and intensities. At low temperatures, immune activity decreases because enzymatic reactions slow down, the production and mobilization of immune cells is reduced, and mechanisms such as chemotaxis and cell proliferation become inefficient, leaving the organism less reactive to pathogens (Wright and Cooper, 1981; Ferguson *et al.*, 2018; Carter *et al.*, 2021). At high temperatures, the immune response can be diminished because proteins and enzymes become destabilized, and some immunological components are suppressed or altered, such as leucocytes and neutrophils, mainly impacting the adaptive immune response (Rollins-Smith, 2017; Weerathunga and Rajapaksa, 2020). In addition, physiological responses to these stressors often differ between the sexes. In anurans, females generally exhibit higher physiological costs associated with reproduction, including oocyte production and, in some species, parental care, which may influence their ability to maintain locomotor performance during physiological challenges (Wells, 1977; Grafe *et al.*, 1992; Hayward and Gillooly, 2011). Sex differences in immune function are also widely documented in vertebrates, including amphibians, and may affect the intensity and duration of inflammatory responses (Nunn *et al.*, 2008; Klein and Flanagan, 2016). Thus, females may be particularly vulnerable to the combination of extreme temperatures and immune activation, with potential consequences for their survival and reproductive success.

The species *Xenopus laevis* provides a relevant model for investigating the influence of different thermal conditions and immune stress on locomotor behaviour. In addition to its widespread use in physiological research, this species is invasive, already present in several regions of the world, and its dispersal capacity, broad thermal tolerance and resistance to pathogens are thought to contribute to its ecological success, with impacts on native invertebrate and vertebrate communities (Lillo *et al.*, 2005; Measey *et al.*, 2012; Measey, 2016; Rödder *et al.*, 2017). Consequently, alterations in locomotor performance, driven by thermal conditions and immune activation, can influence both the dynamics of invasive populations and the persistence of natural populations under ongoing climate change (Miller, 1982; Raffel *et al.*, 2006; Tattersall *et al.*, 2012; Seebacher *et al.*, 2014; Weerathunga and Rajapaksa, 2020). Previous studies have shown that immune activation via LPS reduces locomotor performance in *X. laevis*, with more pronounced effects in females (Oliveira *et al.*, 2024a, 2024b); however, how ambient temperature modulates this response remains uncertain.

In this study, we examined the effects of different temperatures on the locomotor performance of female *X. laevis* subjected to an LPS-induced immune challenge. We tested the hypothesis that inflammatory activation impairs locomotor performance and evaluated how this effect manifests itself at ecologically relevant temperatures, this highlights the ecological and conservation relevance of the effects of thermal and immunological stressors on amphibians.

## Materials and Methods

### Study animals

The 42 juvenile female African clawed frogs (*X. laevis*, Daudin, 1802) used in this study originated from a population of adult individuals captured in the wild at a known invasive hotspot in the Deux-Sèvres department of western France. Subsequently, the captured animals were reared and maintained in captivity for several years at the National Museum of Natural History (MNHN) in Paris, France. The experimental frogs used in this study represent the first generation bred in captivity (F1) derived from founders captured in the wild. The offspring came from different covens (three different males and three different females) and was mixed. All frogs, of both the F0 and F1 generations, were kept in automated water recirculation systems (XenRack, Aquatic Enterprises) under controlled conditions (12:12 h light/dark photoperiod). The ambient and tank temperature was kept constant at 21°C, corresponding to the species’ thermal optimum (Casterlin and Reynolds, 1980; Miller, 1982). The animals were fed twice a week with standardized portions of bovine heart. Although *X. laevis* is native to Southern Africa, it has established invasive populations in several regions of the world due to accidental or intentional releases from laboratory facilities (Lillo *et al.*, 2005; Fouquet and Measey, 2006). The population from western France studied here experiences a temperate climate with marked seasonal variation in water temperature, providing a realistic ecological context for studies of thermal and physiological responses. All experimental procedures were conducted in accordance with institutional ethical guidelines and approved by the Cuvier Committee.

### Pre-experimental treatment and procedures

The experiments were conducted between September and December 2023. Prior to testing, the animals from the F1 generation were removed from their holding tanks and placed individually in uniform containers (width: 27.5 cm × height: 13.5 cm; water depth: 17.0 cm), maintained under the same environmental conditions described above. The frogs were fasted for 24 h before each experimental trial to standardize their metabolic state. Each group of animals (experimental and control) underwent locomotor tests under three different thermal conditions: cold (15°C), optimal (22°C) and warm (27°C). Temperature treatments followed a protocol adapted from Araspin *et al.* (2020), Araspin (2023). The experimental temperatures (15, 22 and 27°C) were selected to represent ecologically relevant thermal conditions experienced by *X. laevis*. The intermediate temperature (22°C) corresponds to the reported thermal optimum for locomotor and physiological performance in anurans, whereas 15 and 27°C represent suboptimal cold and warm conditions commonly encountered in natural aquatic habitats, particularly during seasonal variation and heat waves (Navas, 1996, 1997; Angilletta *et al.*, 2002; Walther *et al.*, 2002; Herrel and Bonneaud, 2012a; Araspin *et al.*, 2020).

Right before experiments frogs were transferred to a temperature-controlled incubator (Aqualytic-LIEBHERR, model TC 256 G, range 2–40°C) set to the target temperature. The animals tested were always placed in the incubator at 7:00 AM for a 3-h thermal equilibration period, in order to allow their body temperature to equalize with the water temperature. After the 3-h acclimation period, the animals received injections of saline solution or LPS, and tests were performed 1 and 24 h after injection. During acclimation, water temperature inside each container was continuously monitored using a type-K thermocouple. The actual temperatures experienced by the animals during the acclimation period were: 15°C treatment: 15.1 ± 0.4°C; 22°C treatment: 22.0 ± 0.3°C; 27°C treatment: 27.2 ± 0.5°C [mean ± standard deviation (SD)].

The temperature of the testing room was adjusted to closely match each experimental temperature to minimize thermal gradients during trials. Locomotor tests were conducted in experimental arenas containing water maintained at the corresponding target temperature (15, 22 or 27°C).

Body mass was recorded before and after each test using an electronic scale (precision: ±0.1 g).

### Experimental design and animal allocation

A total of 42 female *X. laevis* frogs were used in this study, in a mixed experimental design that combined intra-individual and inter-individual comparisons.

Six frogs were randomly assigned to the control group which underwent an injection. These animals served as a reference group for repeated measurements and were tested at all three temperature treatments (15, 22 and 27°C) for the two performance tests (locomotor endurance and jump force). Therefore, each individual in the control group was measured six times (3 temperatures × 2 performance tests). To minimize residual effects and ensure complete physiological recovery, a 7-day interval was imposed between temperature treatments in each performance test. This design allowed us to quantify the effect of temperature on locomotor performance in the absence of immune activation, controlling for individual variation.

The remaining 36 frogs were assigned to the LPS treatment. Since LPS induces a transient, yet intense, systemic inflammatory response, it is known that repeated immune challenges alter subsequent responses and may lead to sensitization or immune suppression. To avoid these confounding effects, each frog injected with LPS was tested only once, under a single temperature condition. This ensured that each performance measurement reflected an innate immune response under a defined thermal environment.

Within the LPS group, animals were randomly assigned to temperature treatments. Eighteen frogs were used to assess locomotor endurance (*n* = 6 per temperature) and an independent set of 18 frogs was used to measure jumping strength (*n* = 6 per temperature). This separation avoided interference between tasks and fatigue effects.

This mixed design provides a robust thermal baseline from healthy control animals, while allowing for unbiased estimation of temperature-dependent immune activation effects without interference from repeated inflammatory stimuli.

All animals were euthanized at the end of the experimental procedures, following approved ethical guidelines.

### LPS injection and control procedure

To simulate an immunological challenge, frogs were injected with LPS derived from *Escherichia coli* serotype O111:B4, purified by phenol extraction (Sigma-Aldrich). The solution was diluted in sterile saline and adjusted to a final dose of 2 mg/kg for each individual, based on body mass. Injections were administered into the dorsal lymph sac using a 29-gauge needle (following Bicego and Branco, 2002; Llewellyn *et al.*, 2011; Olarte, 2017; Oliveira, 2022). All injections were performed at 9:00 AM to standardize circadian effects. Performance tests were conducted at two time points: 1 and 24 h after injection, corresponding to the peak and decline of LPS-induced immune activation. Control animals received an equivalent volume of phosphate-buffered saline (PBS, pH 7.4; Sigma-Aldrich), injected following the same protocol.

### Locomotor performance assays

Locomotor endurance was assessed using a circular aquatic track with a total circumference of 3 m and a water depth of 10 cm. Each frog was placed individually on the track and gently stimulated to swim using tactile stimulation until exhaustion, defined as the inability to resume movement after being turned ventral-up. We recorded the total time spent actively swimming (in minutes) and the distance travelled (in meters) using a stopwatch and manual tracking, following the procedures described by Herrel *et al.* (2012); Herrel and Bonneaud (2012a, 2012b) and Herrel *et al.* (2014). The water temperature was maintained at the designated test condition (15.1 ± 0.4°C; 22°C treatment: 22.0 ± 0.3°C; 27°C treatment: 27.2 ± 0.5°C; mean ± SD), using a Julabo F12-ED heating/cooling system.

Jump force was quantified using a 20 × 10 cm piezoelectric force platform connected to a Kistler charge amplifier (as described in Herrel *et al.*, 2014). Frogs were placed individually on the platform and remained motionless for a few seconds before being gently stimulated to jump. Force signals were sampled at 500 Hz over 60-second recording sessions, during which multiple jumps (typically 3–5) were elicited. Forces along the X, Y and Z axes were recorded using Kistler Bioware software. Signals were drift-corrected when necessary, and the resultant force (in newtons—N) was calculated for each jump. We retained the three highest-force jumps per individual for statistical analysis, following the procedure described by Herrel *et al.* (2014).

**Figure 1:**
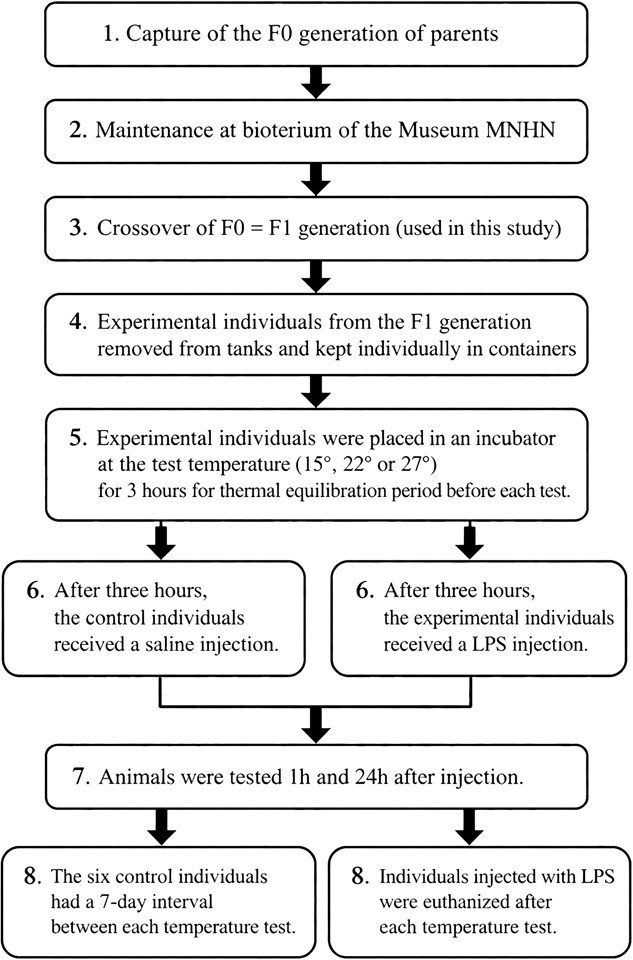
Experimental design. Flowchart summarizing the sequence of procedures, from capture and maintenance of F0 individuals to the use of F1 offspring in experiments. Individuals were acclimated to different temperatures (15, 22 or 27°C) and assigned to control (saline injection) or experimental (LPS injection) groups. Locomotor performance was assessed 1 and 24 h after injection.

The sequence of experimental procedures can be seen in Fig. 1.

### Ethical statement

All experiments conducted were analysed and approved by the ethics committee of the Muséum National d’Histoire Naturelle MNHN (Comité Cuvier).

## Statistical analysis

Data normality was assessed using the Shapiro–Wilk test. When necessary, data were log₁₀ transformed to meet the assumptions of normality and homogeneity of variances.

To assess the influence of body mass on each performance variable, an analysis of covariance was performed with mass as a covariate. Since body mass had no significant effect on any of the response variables (all *P* > 0.05; see Supplementary Table S1), it was excluded from subsequent analyses.

To assess whether locomotor performance differed between time points after injection (1 and 24 h), repeated-measures analyses of variance (ANOVAs) were conducted. Performance traits (endurance and jump force) were treated as dependent variables, and time after injection (1 and 24 h) and treatment (LPS or control) were included as fixed factors, with analyses conducted separately at each test temperature (see Tables S5–S8). To compare performance between the LPS-injected groups and the control groups at each temperature, independent t-tests were used, treating the treatment group as the independent factor and the performance variables as dependent variables (see Table S4).

To assess the effect of temperature on each treatment group (control or LPS), one-way ANOVAs were performed with temperature (15, 22 and 27°C) as the independent factor. Tukey’s *post hoc* tests were applied to determine which temperatures differed significantly.

All analyses were conducted in R version 4.4.2 (R Core Team, 2024). The full script and list of R packages used are provided in Supplementary Material 2.

## Results

### Effect of time post-injection

Since the timing of the injection (1 vs 24 h post-injection) did not significantly influence locomotor performance (all *P* > 0.05; Tables S2 and S3), the data from both time points were pooled for subsequent analyses in order to increase statistical power.

To verify that this approach did not obscure potential temporal differences, all main analyses were also conducted separately for each time point (1 and 24 h post-injection). These included independent comparisons between treatment groups at each temperature and analyses of temperature effects within each treatment group. Results were highly consistent between time points and closely matched those obtained from the pooled dataset (see Supplementary Tables S2–S3), indicating that pooling did not affect the overall patterns or conclusions.

### Locomotor endurance

**Figure 2:**
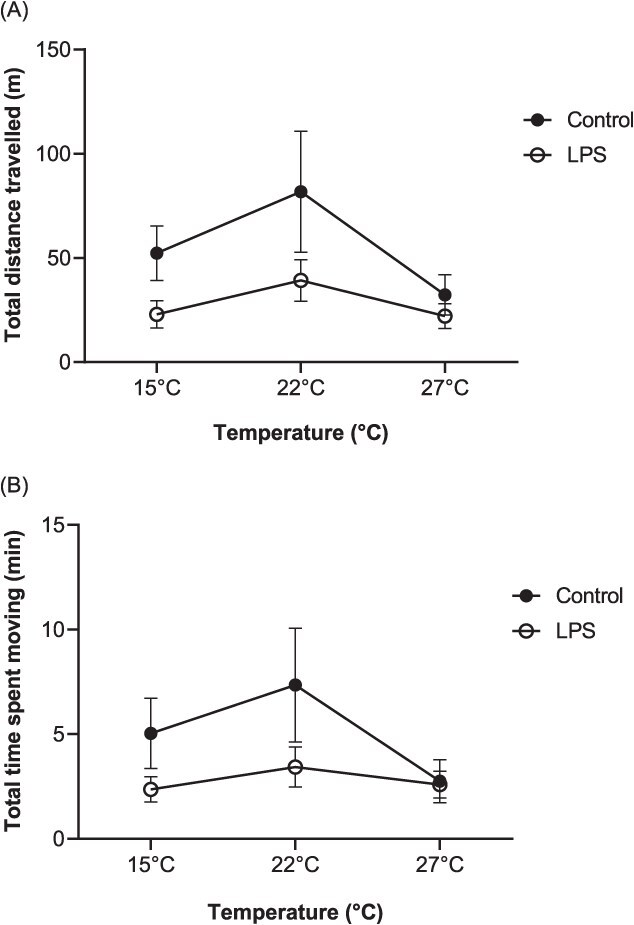
Comparison of locomotor resistance between female *Xenopus laevis* injected with saline solution (control) and the group treated with LPS at three temperatures (15, 22 and 27°C). (A) Average total time spent moving until exhaustion, measured in minutes (min). (B) Average total distance travelled until exhaustion, measured in meters (m). Data are presented as mean ± SD. X-axis: temperatures. Y-axis: variable analysed. Black circles represent the control individuals, injected with saline solution. White circles represent the individuals treated with LPS.

Relative to controls, LPS-injected frogs travelled shorter distances at all test temperatures, reducing total distance by 56% at 15°C (*n* = 6 per group, 12 total; *t*_2.10_ = 5.87, *P* < 0.001), 52% at 22°C (*t*_2.10_ = 3.89, *P* = 0.003) and 32% at 27°C (*t*_2.10_ = 2.35, *P* = 0.041; Table 1 and Fig. 2a). LPS-injected frogs had reduced travel distances at 15 and 27°C compared to 22°C, by 41 and 44%, respectively (*n* = 6 per group, 18 total; *F*_2,15_ = 11.1, *P* < 0.001), with similar performance at thermal extremes (Table S3). Control frogs decreased the distance travelled significantly at thermal extremes relative to 22°C (*n* = 6 per group, 18 total; *F*_2,15_ = 13.1, *P* < 0.001; Table 2), with 36 and 60% reductions at 15 and 27°C, respectively. Distance at 27°C was also 38% shorter than at 15°C (all *P* < 0.05; Table S3).

**Table 1:** Independent sample t-tests comparing locomotor performance (endurance and jump force) between female *Xenopus laevis* injected with saline (control) and LPS-treated group at three temperatures (15, 22 and 27°C)

Comparisons/variables		Mean difference	SE	*df*	*t*	*P*
Control vs LPS—15°C						
*Locomotor endurance*	Total distance covered (m)	29.3	5.0	2.10	5.87	<0.001
	Total time spent moving (min)	2.68	0.67	2.10	3.99	0.003
*Jump force*	Total jump force (N)	1.17	0.36	2.10	3.23	0.009
Control vs LPS—22°C						
*Locomotor endurance*	Total distance covered (m)	42.6	10.9	2.10	3.89	0.003
	Total time spent moving (min)	3.92	1.18	2.10	3.31	0.008
*Jump force*	Total jump force (N)	2.05	0.85	2.10	2.41	0.037
Control vs LPS—27°C						
*Locomotor endurance*	Total distance covered (m)	10.2	4.34	2.10	2.35	0.041
	Total time spent moving (min)	0.16	0.34	2.10	0.48	0.639
*Jump force*	Total jump force (N)	1.02	0.42	2.10	2.37	0.039

Note: *df* = degrees of freedom; SE = standard error difference.

**Table 2:** One-way ANOVAs comparing locomotor performance (endurance and jump force) of female *Xenopus laevis* in the control (saline) and LPS-treated groups at three temperatures (15, 22 and 27°C)

Treatment group/variables	Temperature (°C)		Mean	SD	*df*	*F*	*P*
Control group							
*Locomotor endurance*		15	52.3	10.5			
	Total distance covered (m)	22	81.8	25.5	2.15	13.1	<0.001
		27	32.2	9.48			
		15	5.04	1.46			
	Total time spent moving (min)	22	7.35	2.77	2.15	9.03	0.003
		27	2.76	0.79			
		15	2.87	0.77			
*Jump force*	Total jump force (N)	22	4.65	1.98	2.15	4.62	0.027
		27	2.42	0.94			
LPS group							
*Locomotor endurance*		15	22.9	6.16			
	Total distance covered (m)	22	39.2	8.18	2.15	11.1	<0.001
		27	22.0	4.83			
		15	2.36	0.73			
	Total time spent moving (min)	22	3.43	0.83	2.15	4.32	0.033
		27	2.59	0.27			
		15	1.70	0.43			
*Jump force*	Total jump force (N)	22	2.60	0.64	2.15	8.65	0.003
		27	1.40	0.45			

Note: *df* = degrees of freedom; SD = standard deviation.

Analyses were performed separately for each group.

Time spent moving also declined in LPS-injected frogs relative to controls, with 53% less at 15°C (*t*_2.10_ = 3.99, *P* = 0.003) and 54% less at 22°C (*t*_2.10_ = 3.31, *P* = 0.008; Table 1 and Fig. 2b). No difference was observed at 27°C. In the LPS group, only frogs at 15°C moved less than at 22°C, with a 31% reduction (*F*_2,15_ = 4.32, *P* = 0.033 and 0.034; Table S3), and time spent moving at 15 and 27°C did not differ significantly. Among controls, this variable dropped significantly at 15 and 27°C compared to 22°C (*F*_2,15_ = 9.03, *P* = 0.003; Table 2), with 32 and 62% reductions, respectively. Frogs at 27°C also moved 45% less than at 15°C (*P* < 0.05; Table S3).

### Jump force

**Figure 3:**
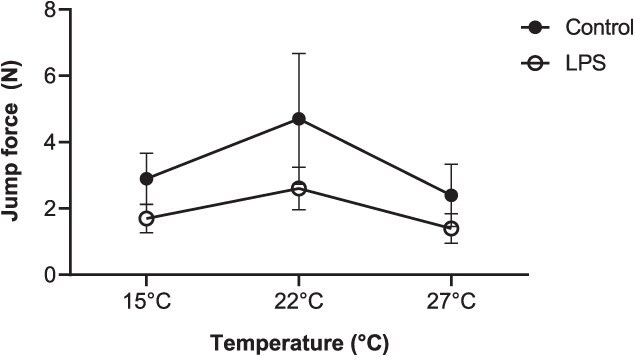
Comparison of average jump force, measured in Newtons (N), between female *Xenopus laevis* injected with saline solution (control) and the group treated with LPS at three temperatures (15, 22 and 27°C). Data are presented as mean ± SD. X-axis: temperatures. Y-axis: variable analysed. Black circles represent control individuals injected with saline solution. White circles represent the individuals treated with LPS.

Frogs injected with LPS showed reduced jump force at all three test temperatures compared to the control group, being 41% lower at 15°C (*n* = 6 per group, 12 total; *t*_2.10_ = 3.23, *P* = 0.009), 45% at 22°C (*t*_2.10_ = 2.41, *P* = 0.037) and 42% at 27°C (*t*_2.10_ = 2.37, *P* = 0.039; Table 1 and Fig. 3). Animals in the experimental group injected with LPS reduced jump force at extreme temperatures compared to 22°C (*n* = 6 per group, 18 total; *F*_2,15_ = 8.65, *P* = 0.003; Table 2), with a 40 and 46% reduction at 15 and 27°C, respectively (Table S3). No differences were observed between the temperature extremes. Animals in the control group also reduced jump force at extreme temperatures compared to the reference temperature of 22°C (*n* = 6 per group, 18 total; *F*_2,15_ = 4.62, *P* = 0.027; Table 2), with a 38% reduction at 15°C and 49% at 27°C, with no significant differences between the extreme temperatures (Table S3).

## Discussion

Our results show that both an immune challenge and temperature influence locomotor performance in *X. laevis*. LPS administration under the three thermal conditions analysed reduced locomotor endurance and jump strength. The magnitude of the LPS effect varied among the temperatures analysed, which suggests a temperature-modulated immune response. For example, LPS reduced locomotor endurance by 56% at 15°C and 52% at 22°C, but only by 32% at 27°C, suggesting a weaker effect at higher temperatures. A similar pattern was observed for the time spent moving, where the effects of LPS were evident at 15 and 22°C, but absent at 27°C. In contrast, jump strength showed a relatively consistent reduction (approximately 41–45%) across all temperatures. Therefore, individuals treated with LPS consistently exhibited lower locomotor endurance than controls at all temperatures for most of the variables analysed. These observations are consistent with the idea that the physiological effects of immune challenge can be modulated by temperature, potentially due to metabolic changes which are dependent on thermal conditions.

The temperature treatments used in this study represent ecologically meaningful thermal conditions for *X. laevis*. The intermediate temperature (22°C) corresponds to the thermal optimum for locomotor and physiological performance commonly reported for this species and other anurans (Navas, 1996, 1997; Angilletta *et al.*, 2002). In contrast, the lower temperature (15°C) represents a suboptimal but ecologically realistic condition, frequently encountered during colder seasons or in deeper or shaded aquatic habitats, where reductions in locomotor performance have been documented (Navas *et al.*, 1999; Herrel and Bonneaud, 2012a). The higher temperature (27°C) reflects upper-range temperatures commonly recorded in shallow water bodies during warm periods and heat waves, conditions that are becoming increasingly prevalent under climate change scenarios (Walther *et al.*, 2002; Araspin *et al.*, 2020). Together, these temperatures span a biologically relevant thermal gradient, allowing the assessment of locomotor performance and immune-related costs under optimal, cold-stress and heat-stress conditions that amphibians are likely to experience in natural environments.

The observed temperature-dependent reduction in locomotor endurance is consistent with the well-established thermal performance curve in anurans, in which locomotion is optimized at intermediate temperatures and declines at thermal extremes (Navas, 1996, 1997; Angilletta *et al.*, 2002; Walther *et al.*, 2002; Butler *et al.*, 2013; Araspin *et al.*, 2020). Contrary to our initial expectations, the most pronounced reduction in locomotor endurance was observed at 15°C rather than at 27°C, indicating that cold temperatures exert a stronger suppressive effect on locomotor performance in immunologically challenged individuals.

It is important to acknowledge that amphibians are ectothermic organisms and that water temperature directly affects locomotor performance, independently of immune activation. All locomotor trials were conducted at the experimental temperatures, and therefore individuals tested at 15°C may have exhibited reduced movement due to direct thermal constraints alone. As a result, the present experimental design does not allow a complete disentanglement of the direct effects of temperature on muscle performance from those mediated by immune activation or energetic trade-offs. This represents a limitation of the study and should be considered when interpreting the reduced locomotor endurance observed at lower temperatures. Despite this limitation, the combined influence of immune challenge and temperature remains biologically relevant, as amphibians in natural environments are frequently exposed to simultaneous thermal variation and immune stressors, such as pathogens and environmental pollutants. Under these conditions, reductions in locomotor endurance may compromise ecologically critical behaviours, including predator avoidance, dispersal, foraging efficiency and reproductive activities, potentially increasing individual vulnerability and reducing population resilience.

At high temperatures, the relatively small difference between control and LPS-treated females suggests that heat stress alone substantially constrains locomotor endurance, potentially masking additional costs associated with immune activation. Elevated temperatures increase metabolic rates and can push ectothermic organisms closer to their locomotor performance limits, reducing locomotor capacity even in the absence of immune activation. Under such conditions, control individuals may already experience substantial reductions in performance, leaving limited scope for additional declines caused by the activation of the immune system. High temperatures are also known to accelerate inflammatory processes and cellular activity (Cossins and Bowler, 1987; Dantzer, 2004; 2006; Lima *et al.*, 2020), but they may simultaneously induce physiological stress, including oxidative damage, neuromuscular impairment and electrolyte imbalance (Cone and Marchalonis, 1972; Cohen *et al.*, 2018). Consequently, both individuals in the control group and those treated with LPS may exhibit reduced locomotor activity, leading to lower metabolic expenditure and heat production, which could explain the similarly low resistance observed at 27°C.

In contrast, cold temperatures appeared to have a less pronounced effect on locomotor endurance in control individuals. Although low temperatures reduce metabolic rates and muscle performance in ectotherms, these effects may not impose the same acute physiological stress observed under high temperatures. However, when combined with immune activation, cold conditions may exacerbate performance limitations. Temperatures below the species-specific thermal optimum are known to suppress immune efficiency and physiological performance in amphibians (Maniero and Carey, 1997; Raffel *et al.*, 2006), potentially prolonging or altering inflammatory responses. As a result, LPS-treated individuals may experience greater functional impairment under cold conditions, leading to the more pronounced reduction in locomotor endurance observed relative to controls.

In contrast to locomotor endurance, the reduction in jump force induced by LPS was similar across temperatures. Reductions in jump force were consistent across temperatures and groups, suggesting that this trait may be more sensitive to general systemic stress than to temperature-specific effects. While control individuals exhibited reduced locomotor endurance at thermal extremes, jump force remained similarly suppressed at both 15 and 27°C, indicating that endurance may be a more temperature-sensitive component of locomotion than force production.

Although previous studies have reported sex-specific differences in locomotor and immune responses in *X. laevis* (Oliveira *et al.*, 2024b), the present study included only females, which prevents comparisons between sexes. Therefore, while higher energetic demands associated with female reproduction may contribute to increased sensitivity to combined thermal and immune stressors, this interpretation remains speculative within the context of this experiment. Future studies incorporating both sexes, as well as direct metabolic measurements, will be essential to clarify the role of sex-specific energetic trade-offs.

From a conservation perspective, our findings underscore the importance of considering interactive effects of temperature and immune challenge when assessing amphibian vulnerability under climate change scenarios. Increasing temperature variability, combined with rising pathogen pressure, may reduce locomotor endurance in natural populations, limiting dispersal capacity and increasing susceptibility to predation and environmental stress. Such functional impairments may have cascading effects on population persistence, particularly in species already exposed to fragmented habitats and thermal extremes.

In conclusion, this study highlights that both high and low temperatures can impair locomotor endurance in *X. laevis*, especially under immune challenge, although through potentially distinct physiological mechanisms. Recognizing the limitations of the experimental design, these results nonetheless emphasize the ecological relevance of temperature–immune interactions and their potential consequences for amphibian performance and conservation in a rapidly changing climate.

## Supplementary Material

Web_Material_coag035

## Data Availability

The files containing the data are stored in the Harvard Dataverse Database and can be accessed at: https://dataverse.harvard.edu/dataverse/harvard or via the doi: https://doi.org/10.7910/DVN/LI4OCR.
