# Supplementary material for: Thermal and immunological stress modulate the locomotor performance of female Xenopus laevis frogs
Source: Conserv Physiol. 2026 Jun 18;14(1):coag035. doi: 10.1093/conphys/coag035 (PMC13278844; doi:10.1093/conphys/coag035)
Supplement: Web_Material_coag035 [file web_material_coag035.zip › Supplementary Material.pdf]

Supplementary Material

Table S1. ANCOVA testing the effect of body mass on locomotor behavior in female *Xenopus laevis* from saline (control) and LPS-treated groups at three temperatures (15, 22, and 27 °C), with analyses conducted separately for each locomotor variable.

**Variables**	**Comparisons**	**Mean Squares**	**d.f**	***F***	***p***
**Locomotor Endurance**					
Total Distance Covered (m)	Body Mass Control x LPS 15°C	0.0023	1.11	0.16	0.694
	Body Mass Control x LPS 22°C	0.0183	1.11	0.82	0.378
	Body Mass Control x LPS 27°C	0.0518	1.11	3.66	0.071
Total Time until Exhaustion (min)	Body Mass Control x LPS 15°C	13.185	1.11	0.74	0.398
	Body Mass Control x LPS 22°C	5.7021	1.11	1.27	0.272
	Body Mass Control x LPS 27°C	17.014	1.11	2.20	0.154
**Jump Force (N)**	Body Mass Control x LPS 15°C	0.0817	1.11	0.09	0.760
	Body Mass Control x LPS 22°C	9.7221	1.11	3.76	0.067
	Body Mass Control x LPS 27°C	0.2670	1.11	0.14	0.703

Note: d.f. = Degrees of freedom; Significance level $p < 0.05$.

Table S2. Repeated measures ANOVA examining the effect of time after injection (1 h vs. 24 h) on locomotor performance (endurance and jump force) in female *Xenopus laevis* injected with LPS (treatment) or saline (control) across three temperatures (15, 22, and 27 °C), performed separately for each locomotor variable.

**Variables**	**Comparisons**	**Mean Squares**	**d.f**	***F***	***p***
Saline Group					
**Locomotor Endurance**					
Total Distance Covered (m)	1 hour – 24 hours 15°C	0.051	1.5	3.50	0.077
	1 hour – 24 hours 22°C	0.010	1.5	0.27	0.615
	1 hour – 24 hours 27°C	0.004	1.5	0.17	0.666
Total Time until Exhaustion (min)	1 hour – 24 hours 15°C	3.11	1.5	1.07	0.328
	1 hour – 24 hours 22°C	7.001	1.5	0.88	0.372
	1 hour – 24 hours 27°C	0.194	1.5	0.15	0.706
**Jump Force (N)**	1 hour – 24 hours 15°C	0.023	1.5	0.01	0.919
	1 hour – 24 hours 22°C	1.300	1.5	0.29	0.603
	1 hour – 24 hours 27°C	0.644	1.5	0.20	0.659
LPS Group					
**Locomotor Endurance**					
Total Distance Covered (m)	1 hour – 24 hours 15°C	0.024	1.5	1.52	0.249
	1 hour – 24 hours 22°C	0.007	1.5	0.60	0.455
	1 hour – 24 hours 27°C	0.015	1.5	1.23	0.296
Total Time until Exhaustion (min)	1 hour – 24 hours 15°C	0.396	1.5	1.00	0.343
	1 hour – 24 hours 22°C	0.140	1.5	0.17	0.714
	1 hour – 24 hours 27°C	0.020	1.5	0.03	0.848
**Jump Force (N)**	1 hour – 24 hours 15°C	0.829	1.5	2.14	0.177
	1 hour – 24 hours 22°C	3.090	1.5	2.70	0.135
	1 hour – 24 hours 27°C	0.105	1.5	0.21	0.653

Note: d.f. = Degrees of freedom; Significance level $p < 0.05$.

Table S3. Tukey post hoc tests performed following one-way ANOVAs to identify significant pairwise differences in female *Xenopus laevis* within the saline (control) and LPS-treated groups across three temperatures (15, 22, and 27 °C), with analyses conducted separately for each group.

**Treatment / Variables**	**Comparisons**	**Mean difference**	**d.f**	***t***	***p* tukey**
Saline Group					
**Locomotor Endurance**					
Total distance covered (m)	22°C – 15°C	29.5	2.15	3.04	0.021
	22°C – 27°C	49.6	2.15	5.09	<0.001
	15°C – 27°C	20.0	2.15	2.89	0.033
Total time spent moving (min)	22°C – 15°C	2.31	2.15	3.14	0.015
	22°C – 27°C	4.59	2.15	4.25	0.002
	15°C – 27°C	2.28	2.15	3.31	0.022
**Jump Force**					
Total jump force (N)	22°C – 15°C	1.78	2.15	2.30	0.027
	22°C – 27°C	2.23	2.15	2.87	0.029
	15°C – 27°C	0.45	2.15	0.57	0.833
LPS group					
**Locomotor Endurance**					
Total distance covered (m)	22°C – 15°C	16.3	2.15	4.31	0.002
	22°C – 27°C	17.1	2.15	4.5	0.001
	15°C – 27°C	0.89	2.15	0.23	0.970
Total time spent moving (min)	22°C – 15°C	1.07	2.15	2.80	0.034
	22°C – 27°C	0.83	2.15	2.18	0.106
	15°C – 27°C	0.23	2.15	0.60	0.819
**Jump Force**					
Total jump force (N)	22°C – 15°C	0.90	2.15	3.01	0.023
	22°C – 27°C	1.20	2.15	3.99	0.003
	15°C – 27°C	0.30	2.15	0.99	0.589

Note: d.f. = Degrees of freedom; Significance level $p < 0.05$.

Table S4. Independent sample t-tests comparing locomotor performance (endurance and jump force) between control and LPS-treated females at each temperature (15, 22, and 27 °C), analyzed separately for 1 h and 24 h post-injection. Analyses were conducted to verify whether pooling data across time points influenced the results.

**Comparisons / Variables**		**Mean differnce**	**S.E**	**d.f**	***t***	***p***
**1hour after injection**						
**Control vs. LPS - 15°C**						
Locomotor Endurance	Total distance covered (m)	0.37	0.05	2.10	4.26	<0.001
	Total time spent moving (min)	0.42	0.09	2.10	4.68	<0.001
Jump force	Total jump force (N)	1.25	0.79	2.10	1.57	0.047
**Control vs. LPS - 22°C**						
Locomotor Endurance	Total distance covered (m)	0.28	0.07	2.10	3.79	0.004
	Total time spent moving (min)	0.27	0.08	2.10	3.34	0.008
Jump force	Total jump force (N)	1.83	0.23	2.10	1.49	0.048
**Control vs. LPS - 27°C**						
Locomotor Endurance	Total distance covered (m)	0.11	0.07	2.10	1.41	0.031
	Total time spent moving (min)	0.10	0.09	2.10	1.17	0.269
Jump force	Total jump force (N)	0.91	0.56	2.10	1.62	0.036
**24 hours after injection**						
**Control vs. LPS - 15°C**						
Locomotor Endurance	Total distance covered (m)	0.34	0.08	2.10	4.25	0.002
	Total time spent moving (min)	0.48	0.08	2.10	5.99	<0.001
Jump force	Total jump force (N)	1.08	0.38	2.10	2.82	0.018
**Control vs. LPS - 22°C**						
Locomotor Endurance	Total distance covered (m)	0.31	0.09	2.10	3.30	0.008
	Total time spent moving (min)	0.36	0.08	2.10	4.15	0.002
Jump force	Total jump force (N)	1.17	0.49	2.10	2.37	0.039
**Control vs. LPS - 27°C**						
Locomotor Endurance	Total distance covered (m)	0.20	0.06	2.10	3.11	0.011
	Total time spent moving (min)	0.10	0.09	2.10	1.15	0.278
Jump force	Total jump force (N)	1.12	0.90	2.10	1.23	0.047

Note: d.f. = Degrees of freedom; S.E = Standard error difference.

Table S5. One-way ANOVAs comparing locomotor performance (endurance and jump force) of female *Xenopus laevis* in the control (saline) group at three temperatures (15, 22, and 27 °C), analyzed separately for 1 h and 24 h post-injection. Analyses were conducted to verify whether pooling data across time points influenced the results.

**Treatment group / Variables**		**Temperature**	**Mean**	**S.D**	**d.f**	***F***	***p***
**Control group 1 hour after injection**							
Locomotor Endurance	Total distance covered (m)	15°C	1.75	0.07	2.15	16.9	<0.001
		22°C	1.93	0.16			
		27°C	1.48	0.14			
	Total time spent moving (min)	15°C	0.67	0.14	2.15	11.5	<0.001
		22°C	0.83	0.17			
		27°C	0.39	0.15			
Jump force	Total jump force (N)	15°C	2.62	1.86	2.15	12.6	0.016
		22°C	4.90	2.80			
		27°C	2.50	1.15			
**Control group 24 hour after injection**							
Locomotor Endurance	Total distance covered (m)	15°C	1.65	0.14	2.15	6.63	0.009
		22°C	1.83	0.19			
		27°C	1.49	0.3			
	Total time spent moving (min)	15°C	0.68	0.15	2.15	8.78	0.003
		22°C	0.83	0.17			
		27°C	0.41	0.20			
Jump force	Total jump force (N)	15°C	3.07	0.70	2.15	3.03	0.048
		22°C	4.27	1.00			
		27°C	2.27	2.12			

Note: d.f. = Degrees of freedom; S.D = Standard deviation.

Table S6. Tukey post hoc tests performed following one-way ANOVAs to identify significant pairwise differences in female *Xenopus laevis* in the control (saline) group at three temperatures (15, 22, and 27 °C), analyzed separately for 1 h and 24 h post-injection.

**Treatment / Variables**	**Comparisons**	**Mean difference**	**d.f**	***t***	***p* tukey**
Saline Group 1 hour after Injection					
**Locomotor Endurance**					
Total distance covered (m)	22°C – 15°C	0.17	2.15	2.23	0.048
	22°C – 27°C	0.44	2.15	5.77	<0.001
	15°C – 27°C	0.27	2.15	3.64	0.008
Total time spent moving (min)	22°C – 15°C	0.16	2.15	1.78	0.015
	22°C – 27°C	0.43	2.15	4.75	<0.001
	15°C – 27°C	0.27	2.15	2.97	0.024
**Jump Force**					
Total jump force (N)	22°C – 15°C	2.28	2.15	1.93	0.016
	22°C – 27°C	2.40	2.15	2.02	0.040
	15°C – 27°C	0.11	2.15	0.09	0.055
Saline Group 24 hour after Injection					
**Locomotor Endurance**					
Total distance covered (m)	22°C – 15°C	0.18	2.15	1.98	0.052
	22°C – 27°C	0.34	2.15	3.64	0.006
	15°C – 27°C	0.15	2.15	1.66	0.053
Total time spent moving (min)	22°C – 15°C	0.15	2.15	1.49	0.021
	22°C – 27°C	0.42	2.15	4.14	0.002
	15°C – 27°C	0.26	2.15	2.64	0.046
**Jump Force**					
Total jump force (N)	22°C – 15°C	1.20	2.15	1.47	0.034
	22°C – 27°C	2.00	2.15	2.44	0.056
	15°C – 27°C	0.80	2.15	0.97	0.061

Note: d.f. = Degrees of freedom; Significance level $p < 0.05$.

Table S7. One-way ANOVAs comparing locomotor performance (endurance and jump force) of female *Xenopus laevis* in LPS-treated group at three temperatures (15, 22, and 27 °C), analyzed separately for 1 h and 24 h post-injection. Analyses were conducted to verify whether pooling data across time points influenced the results.

Treatment group / Variables		Temperature	Mean	S.D	d.f	*F*	*p*
**LPS group 1 hour after Injection**							
Locomotor Endurance	Total distance covered (m)	15°C	1.38	0.10		12.5	<0.001
		22°C	1.64	0.08			
		27°C	1.37	0.12			
	Total time spent moving (min)	15°C	0.25	0.16	2.15	8.07	0.004
		22°C	0.56	0.09			
		27°C	0.26	0.16			
Jump force	Total jump force (N)	15°C	1.37	0.58	2.15	6.98	0.007
		22°C	3.07	1.13			
		27°C	1.58	0.76			
**LPS group 24 hour after Injection**							
Locomotor Endurance	Total distance covered (m)	15°C	1.30	0.13	2.15	7.13	0.007
		22°C	1.52	0.12			
		27°C	1.28	0.08			
	Total time spent moving (min)	15°C	0.19	0.12	2.15	7.05	0.007
		22°C	0.46	0.13			
		27°C	0.30	0.11			
Jump force	Total jump force (N)	15°C	1.98	0.62	2.15	13.5	0.001
		22°C	3.10	0.67			
		27°C	1.15	0.66			

Note: d.f. = Degrees of freedom; S.D = Standard deviation.

Table S8. Tukey post hoc tests performed following one-way ANOVAs to identify significant pairwise differences in female *Xenopus laevis* in the LPS-treated group at three temperatures (15, 22, and 27 °C), analyzed separately for 1 h and 24 h post-injection.

**Treatment / Variables**	**Comparisons**	**Mean difference**	**d.f**	***t***	***p* tukey**
LPS group 1 hour after injection					
**Locomotor Endurance**					
Total distance covered (m)	22°C – 15°C	0.26	2.15	4.21	0.002
	22°C – 27°C	0.27	2.15	4.42	0.001
	15°C – 27°C	0.01	2.15	0.21	0.975
Total time spent moving (min)	22°C – 15°C	0.31	2.15	3.69	0.006
	22°C – 27°C	0.27	2.15	3.21	0.015
	15°C – 27°C	0.04	2.15	0.47	0.885
**Jump Force**					
Total jump force (N)	22°C – 15°C	1.70	2.15	3.43	0.010
	22°C – 27°C	1.48	2.15	2.99	0.023
	15°C – 27°C	0.21	2.15	0.43	0.901
LPS group 24 hour after injection					
**Locomotor Endurance**					
Total distance covered (m)	22°C – 15°C	0.21	2.15	3.14	0.017
	22°C – 27°C	0.23	2.15	3.38	0.011
	15°C – 27°C	0.02	2.15	0.24	0.968
Total time spent moving (min)	22°C – 15°C	0.27	2.15	3.73	0.005
	22°C – 27°C	0.16	2.15	2.23	0.048
	15°C – 27°C	0.10	2.15	1.50	0.320
**Jump Force**					
Total jump force (N)	22°C – 15°C	1.12	2.15	2.96	0.025
	22°C – 27°C	1.95	2.15	5.18	<0.001
	15°C – 27°C	0.83	2.15	2.21	0.101

Note: d.f. = Degrees of freedom; Significance level $p < 0.05$.

```
# ========================================
# R SCRIPT - Thermal and Immune Stress in Frogs
# ========================================

# Install required packages (execute only once)
install.packages(c("car", "multcomp", "ggplot2"))
# Load packages
library(car)
library(multcomp)
library(ggplot2)
# - - - - - - - - - - - - - - - - - - - - - - - - - - - -
# 1. Import data
# - - - - - - - - - - - - - - - - - - - - - - - - - - - -
# Replace "dados.csv" by your actual file name/path
data <- read.csv("dados.csv")
# Check first rows
head(data)
# - - - - - - - - - - - - - - - - - - - - - - - - - - - -
# 2. Normality test (Shapiro-Wilk)
# - - - - - - - - - - - - - - - - - - - - - - - - - - - -
# Assuming your variables are named "Resistance" and "JumpStrength"
shapiro.test(data$Resistance)
shapiro.test(data$JumpStrength)
# - - - - - - - - - - - - - - - - - - - - - - - - - - - -
# 3. Log10 transformation (if needed)
# - - - - - - - - - - - - - - - - - - - - - - - - - - - -
data$Resistance_log <- log10(data$Resistance + 1)
data$JumpStrength_log <- log10(data$JumpStrength + 1)
# - - - - - - - - - - - - - - - - - - - - - - - - - - - -
# 4. ANCOVA: effect of body mass on performance
# ----------------------------------------
# Example for locomotor endurance
ancova_resistance <- lm(Resistance_log ~ mass +
```

```
treatment + factor(temperature), data = data)
anova(ancova_resistance)
# Example for jump force
ancova_jump <- lm(JumpStrength_log ~ mass +
treatment + factor(temperature), data = data)
anova(ancova_jump)
# Display summaries
summary(ancova_resistance)
summary(ancova_jump)
#--------------------------------------------------------
# 5. Independent t-tests for each temperature
#--------------------------------------------------------
# Replace "response" with the variable name you want to test (e.g., "Resistance_log")
t_15 <- t.test(Resistance_log ~ treatment, data = subset(data, temperature == 15))
t_22 <- t.test(Resistance_log ~ treatment, data = subset(data, temperature == 22))
t_27 <- t.test(Resistance_log ~ treatment, data = subset(data, temperature == 27))
# Display results
t_15
t_22
t_27
# - - - - - - - - - - - - - - - - - - - - - - - - - -
# 6. ANOVA for each treatment group
# - - - - - - - - - - - - - - - - - - - - - - - - - - -
anova_control <- aov(Resistance_log ~ factor(temperature),
                     data = subset(data, treatment == "control"))
anova_LPS <- aov(Resistance_log ~ factor(temperature),
                 data = subset(data, treatment == "LPS"))
summary(anova_control)
summary(anova_LPS)
# - - - - - - - - - - - - - - - - - - - - - - - - - - -
# 7. Post-hoc Tukey tests (only if ANOVA is significant)
# - - - - - - - - - - - - - - - - - - - - - - - - - - -
tukey_control <- TukeyHSD(anova_control)
```

```
tukey_LPS <- TukeyHSD(anova_LPS)
# Display results
tukey_control
tukey_LPS
# - - - - - - - - - - - - - - - - - - - - - - - - - - -
# 8. Visualization with ggplot2
# - - - - - - - - - - - - - - - - - - - - - - - - - - -
ggplot(data, aes(x = factor(temperature),
                 y = Resistance_log,
                 fill = treatment)) +
  geom_boxplot() +
  labs(title = "Control vs LPS Comparison at Different Temperatures",
       x = "Temperature (°C)",
       y = "Locomotor Performance (log10-transformed)") +
  theme_minimal().

 - - - - - - - - - - - - - - - - - - - - - - - - - - -

# ==========================================
# R SCRIPT – Separate analyses (1h vs 24h)
# ==========================================

# Load required packages
library(dplyr)

# =========================
# 1. Data organization
# =========================

# Ensure variables are treated as factors
data$Time <- factor(data$Time, levels = c("1h", "24h"))
data$Treatment <- factor(data$Treatment, levels = c("Control", "LPS"))
data$Temp <- factor(data$Temp, levels = c("15", "22", "27"))

# Create subsets for each time point
data_1h  <- subset(data, Time == "1h")
```

```
data_24h <- subset(data, Time == "24h")

# ========================
# 2. T-TESTS (LPS vs Control)
# ========================

cat("===================================\n")
cat("T-TESTS – 1h post-injection\n")
cat("===================================\n")

for(temp in levels(data_1h$Temp)) {
  cat("\nTemperature:", temp, "°C\n")

  # Endurance
  print(t.test(Endurance ~ Treatment,
               data = subset(data_1h, Temp == temp)))

  # Jump Force
  print(t.test(JumpForce ~ Treatment,
               data = subset(data_1h, Temp == temp)))
}

cat("\n===================================\n")
cat("T-TESTS – 24h post-injection\n")
cat("===================================\n")

for(temp in levels(data_24h$Temp)) {
  cat("\nTemperature:", temp, "°C\n")

  # Endurance
  print(t.test(Endurance ~ Treatment,
               data = subset(data_24h, Temp == temp)))

  # Jump Force
  print(t.test(JumpForce ~ Treatment,
               data = subset(data_24h, Temp == temp)))
}

# ========================
# 3. ANOVA – Effect of temperature within each treatment
```

```r
# ========================

cat("\n===================================\n")
cat("ANOVA – 1h post-injection\n")
cat("===================================\n")

# CONTROL – 1h
cat("\nControl – Endurance\n")
summary(aov(Endurance ~ Temp, data = subset(data_1h, Treatment == "Control")))

cat("\nControl – Jump Force\n")
summary(aov(JumpForce ~ Temp, data = subset(data_1h, Treatment == "Control")))

# LPS – 1h
cat("\nLPS – Endurance\n")
summary(aov(Endurance ~ Temp, data = subset(data_1h, Treatment == "LPS")))

cat("\nLPS – Jump Force\n")
summary(aov(JumpForce ~ Temp, data = subset(data_1h, Treatment == "LPS")))



cat("\n===================================\n")
cat("ANOVA – 24h post-injection\n")
cat("===================================\n")

# CONTROL – 24h
cat("\nControl – Endurance\n")
summary(aov(Endurance ~ Temp, data = subset(data_24h, Treatment == "Control")))

cat("\nControl – Jump Force\n")
summary(aov(JumpForce ~ Temp, data = subset(data_24h, Treatment == "Control")))

# LPS – 24h
cat("\nLPS – Endurance\n")
summary(aov(Endurance ~ Temp, data = subset(data_24h, Treatment == "LPS")))

cat("\nLPS – Jump Force\n")
summary(aov(JumpForce ~ Temp, data = subset(data_24h, Treatment == "LPS")))

# ========================
```

```
# 4. Tukey post hoc tests (optional)
# ========================

cat("\n===================================\n")
cat("Tukey post hoc – 1h\n")
cat("===================================\n")

TukeyHSD(aov(Endurance ~ Temp, data = subset(data_1h, Treatment == "Control")))
TukeyHSD(aov(Endurance ~ Temp, data = subset(data_1h, Treatment == "LPS")))

cat("\n===================================\n")
cat("Tukey post hoc – 24h\n")
cat("===================================\n")

TukeyHSD(aov(Endurance ~ Temp, data = subset(data_24h, Treatment == "Control")))
TukeyHSD(aov(Endurance ~ Temp, data = subset(data_24h, Treatment == "LPS")))
```
