# Supplementary figures and images for: Thermal and immunological stress modulate the locomotor performance of female Xenopus laevis frogs
Source: Conserv Physiol. 2026 Jun 18;14(1):coag035. doi: 10.1093/conphys/coag035 (PMC13278844; doi:10.1093/conphys/coag035)

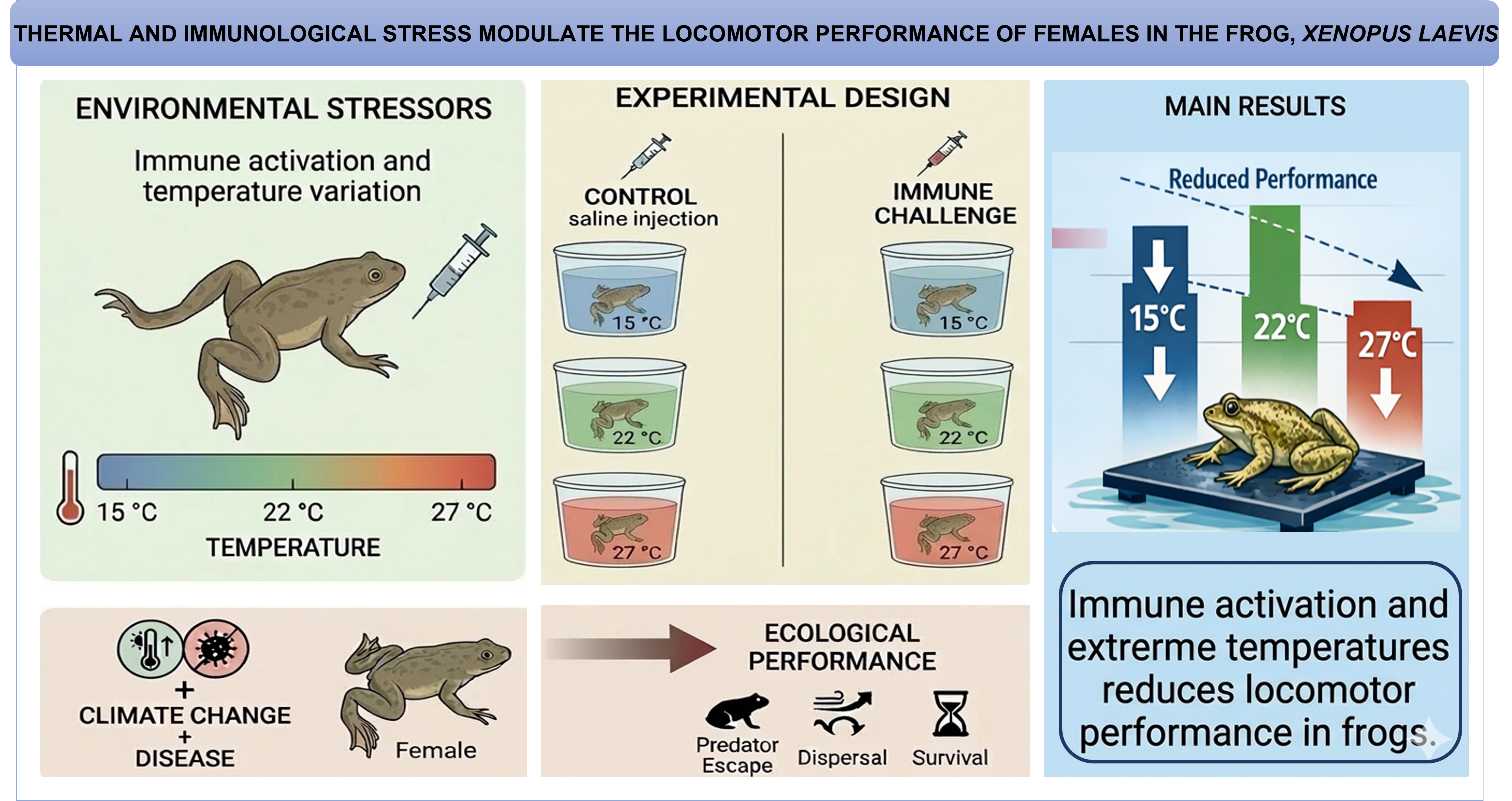

Supplement: Web_Material_coag035 [file web_material_coag035.zip › Graphical Abstract.png]
